# On a Combination of Oximuriatic Gas and Oxigen Gas

**Published:** 1811-10

**Authors:** Humphrey Davy

**Affiliations:** Sec. R. S. Prof. Chem. R. I.


### *On a Combination of Oximuriatic Gas and Oxigen Gas.* *By* HUMPHREY DAVY, Esq. L.L.D. Sec. R. S. Prof. Chem. R. I.

(Phil. Trans. 1811.)

THE oximuriatic gas prepared from manganese, either by mixing it with a muriate and acting upon it by sulphuric acid, or by mixing it with muriatic acid, is, when the oxide of manganese is pure, and whether collected over water or mercury, uniform in its properties; its colour is a pale yellowish green; water takes up about twice its volume, and scarcely gains any colour; the metals burn in it readily; it combines with hydrogen without any deposition of moisture; it does not act on nitrous gas, or muriatic acid, or carbonic oxide, or sulphureous gasses, when they have been carefully dried.

The gas produced by the action of muriatic acid on the salts which have been called hyperoximuriates, on the contrary, differs very much in its properties, according as the manner in which it is prepared and collected is different.

When much acid is employed to a small quantity of salt, and the gas is collected over water, the water becomes tinged of a lemon colour; but the gas collected is the same as that procured from manganese.

When the gas is collected over mercury, and is procured from a weak acid, and from a great excess of salt, by a low heat, its colour is a dense tint of brilliant yellow green, and it possesses properties entirely different from the gas collected over water.

It sometimes explodes during the time of its transfer from one vessel to another, producing heat and light, with an expansion of volume; and it may be always made to explode by a very gentle heat, often by that of the hand*.

---

* My brother, Mr. J. Davy, from whom I receive constant and able assistance in all my chemical inquiries, had several times observed explosions, in transferring the gas from hyperoximuriate of potass, over mercury, and he was inclined to attribute the phænomenon to the combustion of a thin film of mercury, in contact with a globule of gas. I several times endeavoured to produce the effect, but without success, till an acid was employed for the preparation of the gas, so diluted as not to afford it without the assistance of heat. The change of colour and

It is a compound of oximuriatic gas and oxigen, mixed with some oximuriatic gas. This is proved by the results of its spontaneous explosion. It gives off, in this process, from $\frac{1}{6}$ to $\frac{1}{5}$ its volume of oxigen, loses its vivid colour, and becomes common oximuriatic gas.

I attempted to obtain the explosive gas in a pure form, by applying heat to a solution of it in water, but in this case there was a partial decomposition; and some oxigen was disengaged, and some oximuriatic gas formed. Finding that, in the cases when it was most pure, it scarcely acted upon mercury, I attempted to separate the oximuriatic gas with which it is mixed, by agitation in a tube with this metal; corrosive sublimate formed, and an elastic fluid was obtained, which was almost entirely absorbed by $\frac{1}{4}$ of its volume of water.

This gas in its pure form is so easily decomposable, that it is dangerous to operate upon considerable quantities.

In one set of experiments upon it, a jar of strong glass, containing 40 cubical inches, exploded in my hands with a loud report, producing light; the vessel was broken, and fragments of it were thrown to a considerable distance.

I analysed a portion of this gas, by causing it to explode over mercury in a curved glass tube, by the heat of a spirit lamp.

The oximuriatic gas formed was absorbed by water; the oxigen was found to be pure, by the test of nitrous gas.

Fifty parts of the detonating gas, by decomposition, expanded so as to become sixty parts. The oxigen, remaining after the absorption of the oximuriatic gas, was about twenty parts. Several other experiments were made, with similar results. So that it may be inferred, that it consists of two in volume of oximuriatic gas, and one in volume of oxigen; and the oxigen in the gas is condensed to half its volume. Circumstances conformable to the laws of combination of gaseous fluids, so ably illustrated by Mr. Gay-Lussac, and to the theory of definite proportions.

I have stated on a former occasion, that approximations to the numbers representing the proportions in which oxigen and oximuriatic gas combine, are found in 7·5 and 32·9. And this compound gas contains nearly these quantities*.

---

and expansion of volume, when the effect took place, immediately convinced me, that it was owing to a decomposition of the gas.

* In page 245 of the Phil. Trans. for 1810, I have mentioned, that the specific gravity of oximuriatic gas is between 74 and 75 grains per 100 cubical inches. The gas that I weighed was collected over water, and procured from hyperoximuriate of potass, and at that time I conceived that this elastic fluid did not differ from the oximuriatic gas from

The smell of the pure explosive gas somewhat resembles that of burnt sugar, mixed with the peculiar smell of oximuriatic gas. Water appeared to take up eight or ten times its volume; but the experiment was made over mercury, which might occasion an error, though it did not seem to act on the fluid. The water became of a tint approaching to orange.

When the explosive gas was detonated with hydrogen equal to twice its volume, there was a great absorption, to more than $\frac{1}{3}$, and solution of muriatic acid was formed; when the explosive gas was in excess, oxigen was always expelled, a fact demonstrating the stronger attraction of hydrogen for oximuriatic gas than for oxigen.

I have said that mercury has no action upon this gas in its purest form at common temperatures. Copper and antimony, which so readily burn in oximuriatic gas, did not act upon the explosive gas in the cold: and when they were introduced into it, being heated, it was instantly decomposed, and its oxigen set free; and the metals burnt in the oximuriatic gas.

When sulphur was introduced into it, there was at first no action, but an explosion soon took place: and the peculiar smell of oximuriate of sulphur was perceived.

Phosphorus produced a brilliant explosion, by contact with it in the cold, and there were produced phosphoric acid and solid oximuriate of phosphorus.

Arsenic introduced into it did not inflame; the gas was made to explode, when the metal burnt with great brilliancy in the oximuriatic gas.

Iron wire introduced into it did not burn, till it was heated so as to produce an explosion, when it burnt with a most brilliant light in the decomposed gas.

Charcoal introduced into it ignited, produced a brilliant flash of light, and burnt with a dull red light, doubtless owing to its action upon the oxigen mixed with the oximuriatic gas.

It produced dense red fumes when mixed with nitrous gas, and there was an absorption of volume.

When it was mixed with muriatic acid gas, there was a gradual diminution of volume. By the application of heat

---

manganese, except in being purer. It probably contained some of the new gas; for, I find that the specific gravity of pure oximuriatic gas from manganese and muriatic acid is to that of common air, as 244 to 100. Taking this estimation, the specific gravity of the new gas will be about 238, and the number representing the proportion in which oximuriatic gas combines, from this estimation, will be rather higher than is stated above.

the the absorption was rapid, oximuriatic gas was formed, and a dew appeared on the sides of the vessel.

These experiments enable us to explain the contradictory accounts that have been given by different authors of the properties of oximuriatic gas.

That the explosive compound has not been collected before is owing to the circumstance of water having been used for receiving the products from hyperoximuriate of potass, and unless the water is highly saturated with the explosive gas, nothing but oximuriatic gas is obtained; or to the circumstance of too dense an acid having been employed.

This substance produces the phenomena, which Mr. Chenevix, in his able paper on oximuriatic acid, referred to the hyperoxigenised muriatic acid; and they prove the truth of his ideas respecting the possible existence of a compound of oximuriatic gas and oxigen in a separate state.

The explosions produced in attempts to procure the products of hyperoximuriate of potass by acids are evidently owing to the decomposition of this new and extraordinary substance.

All the conclusions which I have ventured to make respecting the undecompounded nature of oximuriatic gas, are, I conceive, entirely confirmed by these new facts.

If oximuriatic gas contained oxigen, it is not easy to conceive, why oxigen should be afforded by this new compound to muriatic gas, which must already contain oxigen in intimate union. Though, on the idea of muriatic acid being a compound of hydrogen and oximuriatic gas, the phenomena are such as might be expected.

If the power of bodies to burn in oximuriatic gas depended upon the presence of oxigen, they all ought to burn with much more energy in the new compound; but copper, and antimony, and mercury, and arsenic, and iron, and sulphur have no action upon it, till it is decomposed; and they act then according to their relative attractions on the oxigen, or on the oximuriatic gas.

There is a simple experiment, which illustrates this idea; Let a glass vessel containing brass foil be exhausted, and the new gas admitted, no action will take place; throw in a little nitrous gas, a rapid decomposition occurs, and the metal burns with great brilliancy.

Supposing oxigen and oximuriatic gas to belong to the same class of bodies; the attraction between them might be conceived very weak, as it is found to be, and they are easily separated from each other, and made repulsive, by a very low degree of heat.

The most vivid effects of combustion known are those produced duced by the condensation of oxigen or oximuriatic gas; but in this instance, a violent explosion with heat and light are produced by their separation, and expansion, a perfectly novel circumstance in chemical philosophy.

This compound destroys dry vegetable colours, but first gives them a tint of red. This and its considerable absorbability by water would incline one to adopt Mr. Chenevix's idea, that it approaches to an acid in its nature. It is probably combined with the peroxide of potassium in the hyper-oximuriate.

That oximuriatic gas and oxigen combine and separate from each other with such peculiar phenomena, appears strongly in favour of the idea of their being distinct, though analogous species of matter. It is certainly possible to defend the hypothesis, that oximuriatic gas consists of oxigen united to an unknown basis; but it would be possible likewise to defend the speculation, that it contains hydrogen.

Like oxigen it has not yet been decomposed; and I some time ago made an experiment, which, like most of the others I have brought forward, is very adverse to the idea of its containing oxigen.

I passed the solid oximuriate of phosphorus in vapour, and oxigen gas together through a green glass tube heated to redness.

A decomposition took place, and phosphoric acid was formed, and oximuriatic gas was expelled.

Now, if oxigen existed in the oximuriate of phosphorus, there is no reason why this change should take place. On the idea of oximuriatic gas being undecompounded, it is easily explained. Oxigen is known to have a stronger attraction for phosphorus than oximuriatic gas has, and consequently ought to expel it from this combination.

As the new compound in its purest form is possessed of a bright yellow green colour, it may be expedient to designate it by a name expressive of this circumstance, and its relation to oximuriatic gas. As I have named that elastic fluid chlorine, so I venture to propose for this substance the name euchlorine, or euchloric gas from ευ and χλωϱος. The point of nomenclature I am not, however, inclined to dwell upon. I shall be content to adopt any name, that may be considered as most appropriate by the able chemical philosophers attached to this Society.

*A Chemical*

